# Acute-on-Chronic Liver Failure From Chronic-Hepatitis-B, Who Is the Behind Scenes

**DOI:** 10.3389/fmicb.2020.583423

**Published:** 2020-12-07

**Authors:** Qian Li, Jun Wang, Mengji Lu, Yuanwang Qiu, Hongzhou Lu

**Affiliations:** ^1^Department of Infectious Diseases, Shanghai Public Health Clinical Center, Shanghai, China; ^2^Center of Clinical Laboratory, The Fifth People’s Hospital of Wuxi, Jiangnan University, Wuxi, China; ^3^Institute of Virology, University Hospital of Essen, University of Duisburg-Essen, Essen, Germany; ^4^Department of Hepatology, The Fifth People’s Hospital of Wuxi, Jiangnan University, Wuxi, China

**Keywords:** ACLF, HBV—hepatitis B virus, B cells, inflammation, cytokines storm

## Abstract

Acute-on-chronic liver failure (ACLF) is an acute syndrome accompanied with decompensation of cirrhosis, organ failure with high 28-day mortality rate. Systemic inflammation is the main feature of ACLF, and poor outcome is closely related with exacerbated systemic inflammatory responses. It is well known that severe systemic inflammation is an important event in chronic hepatitis B (CHB)-ACLF, which eventually leads to liver injury. However, the initial CHB-ACLF events are unclear; moreover, the effect of these events on host immunity as well as that of immune imbalance on CHB-ACLF progression are unknown. Here, we investigate the initial events of ACLF progression, discuss possible mechanisms underlying ACLF progression, and provide a new model for ACLF prediction and treatment. We review the characteristics of ACLF, and consider its plausible immune predictors and alternative treatment strategies.

## Introduction

Acute-on-chronic liver failure (ACLF) is defined as the exacerbation of chronic liver disease initiated by a precipitating event, typically resulting in elevated short-term mortality (Alam et al., 2017). Liver complications or decompensation and rapid disease progression lead to high organ failure risk (45%) and high short-term mortality (90%) (Alam et al., 2017). Acute liver failure (ALF) usually occurs following a precipitating event in the context of chronic liver diseases. In Western countries, alcoholism induced cirrhosis is the main reason of ALF (50–70%). However, in Asia countries, hepatitis B virus (HBV) infection is the main cause of ALF (70%) (Sarin et al., 2009). And HBV-related ALF occurs after acute HBV infection or during chronic HBV infection (Oketani et al., 2014; Manka et al., 2016). The primary cause of ALF is an acute exacerbation of chronic hepatitis B (CHB) infection, which accounts for more than 80% of all cases in China (You et al., 2013). ALF caused by CHB is known as chronic hepatitis B-related ACLF (CHB-ACLF).

CHB-ACLF presents with a poor prognosis because antiviral therapy cannot improve short-term survival, but it can control HBV DNA levels. Liver transplantation (LT) is thought to be an effective treatment approach, but it is not always feasible owing to the lack of liver donors and high cost (Cui et al., 2010; Finkenstedt et al., 2013). Therefore, there is a need for early indicators that can predict CHB progression and acute exacerbation to ACLF. The most effective approach to reduce morbidity and mortality may be to elucidate CHB-ACLF progression and to initiate a suitable therapy at an early stage.

In naturally resolved HBV-infected individuals, synergistic aspects of the immune response can function to efficiently repress residual virus. However, the virus typically persists in cases of CHB due to impaired immunity (Hui et al., 2005; Tan et al., 2015). Immune dysregulation is thought to be involved in the progression of CHB to CHB-ACLF, which involves systemic inflammation and exacerbation of the innate immune system resulting in a cytokine storm (Wu et al., 2018). However, relatively less is known about how impaired host immunity triggers the initiation of CHB-ACLF pathogenesis.

In this review, we emphasize on the mechanisms underlying immune pathogenesis in CHB-ACLF, including new insights into virus-induced immune dysfunction and systemic inflammation interaction with immune exhaustion during final liver damage and multiple organ failure. Finally, we will explore strategies for detecting early indicators of CHB-ACLF and immunotherapy as a treatment approach.

## What Is the Original Trigger Controlling the Switch From Chronicity to Acute Exacerbation?

**FIGURE 1:**
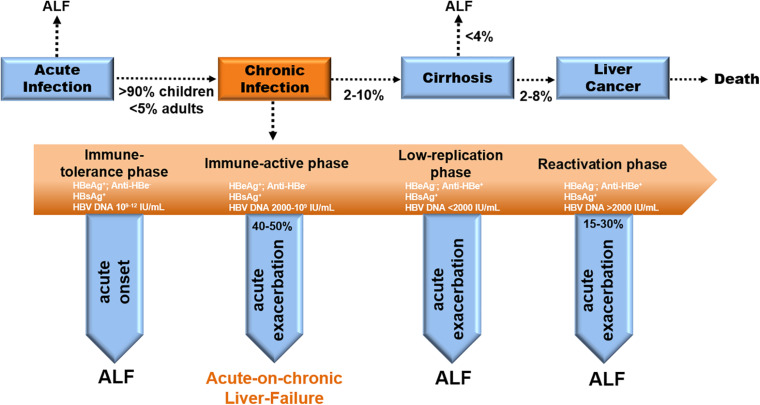
Natural history and disease progression in CHB patients. Evolution to CHB occurs in > 90% of children and < 5% of adults with acute HBV infection. Progression to cirrhosis from CHB appears to be 2–10% per year. The incidence of ALF from cirrhosis is less than 4% yearly. The rate of cirrhosis to liver cancer is 2–8% annually (EASL Jury, 2003). ACLF occurs in the IA phase (40–50%) during chronic infection (Sheen et al., 1985; Lok et al., 1987).

CHB is distinguished by 4 stages in clinical—immune tolerant (IT), immune clearance/active (IA), inactive carrier (IC), and Hepatitis-B-Virus e-Antigen (HBeAg)-negative hepatitis (ENH) (EASL Jury, 2003; Fattovich et al., 2008). Acute severe exacerbation occurs either in the IA phase (40–50%) or in the ENH phase (15–30%) (Figure 1; Sheen et al., 1985; Lok et al., 1987). Notably, CHB-ACLF is a dynamic process ranging from the chronically active phases (IA and ENH) to the acute exacerbation phase that culminates in liver failure. Precipitating factors reported in ACLF include sepsis, alcoholism, and relapse of chronic viral hepatitis, but 40–50% of CHB-ACLF cases have no identifiable triggers (Hernaez et al., 2017).

### Is Innate Immune Recognition the Original Trigger?

Early pathogen detection generally occurs via the recognition of Pathogen-Associated Molecular Patterns (PAMPs) by Pathogen Recognition Receptors (PRRs). The known PRRs include nucleotide-binding oligomerization domain-containing protein (NOD)-like, retinoic acid-inducible gene I (RIG)-like, toll-like receptor (TLR), DNA-sensing receptors and C-type Lectin (Akira et al., 2006; Kawai and Akira, 2009; Takeuchi and Akira, 2010; Pandey et al., 2014). These immunity sensors are distributed across various liver cell types and intrahepatic liver endothelial cells (Ding et al., 2016; Faure-Dupuy et al., 2018). After PAMP recognition by their corresponding PRRs, downstream signaling pathways are sequentially activated involving the expression of various adaptor molecules [e.g., myeloid differentiation primary response gene 88 (MyD88) and TIR-domain-containing adapter-inducing interferon-β (TRIF)], kinase activation or phosphorylation [e.g., TANK binding kinase 1 (TBK1)/transforming growth factor-β activated kinase 1 (TAK1)]. Thereafter, transcription factors are expressed [e.g., nuclear factor kappa-B (NF-κB), interferon regulatory transcription factors (IRFs), c-fos/c-jun, etc.]. This process ultimately leads to effector gene expression including interferon-stimulated-genes (ISGs), NF-κB-inducible genes, and pro-inflammatory genes (IL-2, IL-12, IL-16, etc.) (Kumar et al., 2009, 2011; Kawai and Akira, 2011; Pandey et al., 2014).

The role of TLRs in CHB is debatable. Due to technical limitations, the entirety of the dynamic innate immune response elicited *in vivo* is unknown. It was initially believed that HBV activates TLR2 signaling *in vitro* (Cooper et al., 2005). However, it has been shown that synthesized HBV capsid/core antigen (HBcAg) could be polluted with lipopolysaccharide (LPS)-like ligands in bacteria (Vanlandschoot et al., 2007). In contrast, HBV components are now widely believed to block TLR signaling at various levels. It has been suggested that HBeAg can bind to the co-adaptor of Myd88 and TIRAP, to interfere with TLR2 signaling (Lang et al., 2011). And HBsAg could inhibit TLR2 pathway activation by binding the c-Jun N-terminal protein kinase (JNK) (Wang et al., 2013). Sufficient evidence has demonstrated that HBV is not recognized by TLRs during entry, suggesting that HBV is a “stealth virus” in the early phase of infection (Wieland et al., 2004).

The role of TLRs in CHB-ACLF is also debatable. TLR4 mRNA levels have been reported to be up-regulated in the peripheral blood mononuclear cells (PBMCs) of ACLF patients compared to those in healthy individuals and CHB patients. TLR4 expression in both CD4 + and CD8 + T cells from PBMCs was significantly increased and positively correlated with liver injury severity in ACLF patients, indicating that TLR4 plays a vital role in disease progression (Xu et al., 2015). Xu et al. (2017) found a significant increase in TLR2/4/6/8 expression in the PBMCs of the early stage ACLF patients compared to that in CHB patients. Since TLR2 forms the heterodimers TLR1 and TLR6 to recognize bacterial LPS, it was assumed that TLR up-regulation in the PBMCs of ACLF patients represented the secondary recognition of bacteria from the circulation or gut (Xu et al., 2017). However, Wang et al. (2010) reported that the mRNA levels of TLR3/5/7/9/10 were down-regulated, and the protein level of TLR7 was confirmed to be decreased in CHB-ACLF patients compared to that in CHB patients in the IA phase. Since CHB-ACLF is a dynamic disease, patient data originating from different phases of the disease lead to different TLR recognition. These results suggest that TLR expression varies among different CHB-ACLF stages. However, there is no definite evidence regarding whether TLR recognition triggers the progression from CHB to acute CHB-ACLF exacerbation.

Evidence suggests that strong HBV replication may be sensed by the innate immune system (Durantel and Zoulim, 2009). For example, *ex vivo* experiments have proven that HBV replication in HepaRG cell lines activates some ISGs (Lucifora et al., 2010). RIG1 and MDA5 are the primary DNA sensors that are also involved in inducing low chronic inflammation (Safari-Arababadi et al., 2019). Additionally, *in vivo* experiments demonstrated some activation of IFN-α stimulated genes in HBV-infected hepatocytes in chimeric mice (Lutgehetmann et al., 2011). The mechanism by which HBV is sensed in infected cells is likely mediated by the growing family of PRRs capable of discriminating intracellular pathogen DNA from the host’s DNA (Sharma et al., 2015). Interestingly, it has been reported that HBV does not interfere with the innate immune response in the human liver because HBV patient liver specimens fail to induce ISG expression following TLR3 treatment (Suslov et al., 2018). However, subsequent studies have refuted this hypothesis because the model of a pre-existing long-standing infection is not ideal (Vyas et al., 2018). It is generally accepted that the weak IFN response serves as a strategy to enable HBV to escape innate immune recognition (Wieland and Chisari, 2005).

The IFN response is also suppressed in the exacerbation phase of CHB-ACLF as indicated by *in vitro* experiments demonstrating that SOCS3, a well-known IFN signaling suppressor, was significantly elevated in the liver and PBMCs of a mouse model of CHB-ACLF (Li et al., 2014). This result supports the hypothesis that SOCS3 is involved in immune homeostasis in pathological conditions by negatively regulating cytokine or hormone signaling (Carow and Rottenberg, 2014). However, the newly identified interferon-gamma induced protein (IFI16) is significantly more highly expressed in ACLF patients than in CHB patients and healthy individuals (Kerur et al., 2011; Connolly and Bowie, 2014). In contrast to the weak IFN response observed in CHB patients, the high IFN response in the ACLF patient livers indicates that selective IFI16 recruitment in the liver may initiate a destructive immune response leading to liver injury (Pang et al., 2018). Interestingly, this study was performed over a late time course of liver damage, which is clinically diagnosed as high-grade liver failure (> 2).

The hypothesis that TLR recognition initiates the transition from CHB to ACLF has been refuted due to the lack of a defined turning point from chronicity to the acute exacerbation phase of CHB-ACLF and the limited observations regarding the relationship between TLR recognition, the IFN response, and the prognosis of CHB-ACLF patients.

### Serum Viral Load of CHB-ACLF Patients Does Not Differ From That of CHB Patients

In CHB patients, the IT phase is characterized by high HBV DNA levels but minimal liver damage as detected by histological analysis (Andreani et al., 2007). From the data of other patient cohorts, it can be observed that the serum HBV DNA load in ACLF patients is lower than that in CHB patients (Table 1; Gao et al., 2017; Lei et al., 2019). In addition, other serum parameters such as serum HBsAg level are lower in CHB-ACLF patients than in CHB patients. Furthermore, the serum HBeAg level is lower in CHB-ACLF patients than in HBeAg-positive CHB patients (Table 1; Zhang Y.M. et al., 2014).

**TABLE 1:** Serum virus load in ACLF and different stage of CHB.

**Variables**	**ACLF**	**Immune tolerance**	**Immune active**	**Low replication**	**Reactivation**	**References**
HBeAg serostatus	+/−	+	+	−	−	Andreani et al., 2007
HBV DNA (log IU/mL)	2.2–7.3	8.0–8.6	4.5–9.0	~−3.6	2.9–6.2	Andreani et al., 2007
HBsAg (log IU/mL)	2.74–4.03	4.54–4.97	3.67–4.43	Unknown	Unknown	Lei et al., 2019
ALT UNL	23–2,347	20–33	27–353	14–48	20–106	Andreani et al., 2007

*+, with; −, without.*

It is reasonable to assume that innate immune recognition like via TLRs cannot induce CHB progression to CHB-ACLF. It is essential to explore and uncover the potential triggers to achieve early ACLF indicators because the serological characteristics of CHB are similar to those of CHB-ACLF.

## Potential Triggers of CHB-ACLF

The potential triggers of CHB-ACLF were widely researched. Bacterial infections and alcoholism were found to be the two major identifiable factors in the CANONIC study, while hepatitis B relapse was found to be the predominant factor followed by bacterial infections in studies conducted in China (Moreau et al., 2013; Shi et al., 2015). The CANONIC criteria primarily identified hepatic-ACLF triggers categorized as liver toxins (alcohol, hepatitis) and extrahepatic-ACLF triggers (e.g., infections). Despite exhaustive examination, the trigger for CHB-ACLF remains unknown in 20–45% of cases, particularly for cases in China (Baumert et al., 1996). We speculate that the potential triggers among these CHB-ACLF patients are related to HBV characteristics such as HBV genotype and mutations (Table 2).

**TABLE 2:** Potential triggers of HBV-ACLF.

**Triggers**	**Specific examples**
HBV genotype	CHB of genotype B with BCP/PC mutations > CHB of genotype C with BCP/PC mutations
HBV viral mutations	• Nucleotide mutations in core promoter and pre-core regions • Amino acid mutations in HBsAg (large HBsAg mutation in I161 and N177 in genotype C), large HBxAg (mutations in K130, V131, and C143), and HBcAg (mutations in E113, T114, A131, and P135)
Host immune pressure	Highly mutated HBcAg led to the corresponding somatic mutations within the variable heavy chain (VH) gene and lacking binding affinity to hepatitis B core antibody (HBcAb)

### HBV Genotype

HBV is classified into 8 genotypes (A–H) by viral characteristics, geographical distribution, and clinical outcomes. CHB patients with genotype B with basal core promoter/pre-core (BCP/PC) mutations were reported more susceptible to develop ALF compared to patients with genotype C with wild-type BCP/PC regions (Baumert et al., 1996; Tong et al., 2005; Kay and Zoulim, 2007). Another report also indicated that patients infected with HBV/Bj are more frequently to develop ALF than patients with HBV/Ae (Ozasa et al., 2006).

### HBV Viral Mutations

Mutations in the core promoter and pre-core (PC) regions have been found to enhance HBV replication *in vitro*, and the PC mutation inhibits HBeAg translation, which may enable the escape from immune attacks on the infected hepatocytes. Higher G1896A PC mutation and BCP double mutation (A1762T/G1764A) occurred in ALF patients than in patients with acute HBV infection (Kosaka et al., 1991; Liang et al., 1991; Sato et al., 1995; Inoue et al., 1998; Friedt et al., 1999; Ozasa et al., 2006). Additionally, single mutations in the BCP/PC region, including C1766T, T1753V (C/A/G), G1862T, T1768A, and G1899A have been shown to be related to elevated HBV replication in some cases with ALF (Liang et al., 1991; Hou et al., 2002; Parekh et al., 2003; Wai et al., 2005). Xu et al. (2011) found CHB patients with BCP/PC mutant are more likely to have ACLF than patients without such a mutation. Strong evidence demonstrated that the A1846T and C1913A mutations are positively associated with the presentation of severe liver diseases (Zang et al., 2018). However, the traditional technique utilized for HBV virus sequencing is PCR amplification, which targets a limited and select number of clones. Additionally, there is limited evidence regarding how HBV mutations in the BCP and PC induce the immune response. HBeAg, a secreted soluble protein, can suppress seroconversion to anti-HBcAg antibody production in some mouse strains, and it could serve as a regulator (Chen et al., 2004). HBeAg is typically present along with HBcAg in HBV infections, but BCP mutations in HBeAg can cause a loss-of-function phenotype (called a basal-core mutation). HBeAg is not synthesized in these conditions, and the lack of its immunomodulatory functions is thought to induce an uncontrolled and enforced immune response to HBcAg that is associated with ALF in some cases (Fagan et al., 1986; Liang et al., 1991).

Unlike the indirect immune dysregulation caused by HBcAg, amino acid mutations in HBsAg, large HBxAg, and HBcAg have been found to induce immune dysfunction. In large HBsAg, I161 and N177 exhibit higher variation frequencies in ACLF patients than in acute hepatitis B patients of genotype C. Meanwhile, in HBxAg, V131, K130, and C143 have exhibited the highest frequency of variation in ACLF patients compared to that in CHB patients (40% of clones for 2/5 patients) with genotype C (Yang et al., 2015). Zhang et al. also found that A131, T114, E113, and P135 in HBcAg had more variation frequency in ACLF patients with both genotype B/C. Additionally, numerous deletion mutations in different clones from base 1997 to 2279 resulted in frameshift mutations in ACLF patients, which introduced a premature stop codon and produced a truncated HBcAg (Yang et al., 2015). However, the sequence read length (<500 bp) obtained here using next-generation sequencing does not cover the complete HBV genome length. Additionally, patients enrolled for research studies are limited. Since HBV genome quasispecies of all individuals were not longitudinally analyzed, the selective pressure of the immune response caused by mutant viral antibodies is unclear (Yang et al., 2015).

Therefore, there is a need for studies on the immune pressure of mutant viral Ag, especially for HBcAg. Mutations or deletions at L60–I97 of HBcAg have been widely studied as T- or B-cell epitopes in ACLF patients, much more than that in IT and acute hepatitis B patients (Alexopoulou et al., 2009; Chen et al., 2010). Other studies have shown accumulated mutations within HBc141-149 epitope in HBV-infected individuals. HBc141-149 is an HLA-A2-restricted epitope can be naturally processed in HBV-infected individuals, and evoke antiviral T cell response in HLA-A2.1/HBV transgenic mice (Sun et al., 2014). Chen et al. compared genetic and functional difference of the virus and host immunity in the liver tissue from chimpanzees of HBV-associated ALF to those with acute hepatitis B. In contrast to acute hepatitis B, HBV strains displayed highly mutated HBcAg, lacked corresponding somatic mutations in heavy chain variable region (VH) genes, and exhibited low binding affinity to HBcAb, ultimately leading to increased HBcAg expression *ex vivo* independent of viral replication levels in the liver of ALF (Chen et al., 2018).

### Are T-Cell Independent B Cell Response, the Driver of ACLF?

Since innate immune recognition lacks specificity and the virological parameters fail to change in ACLF patients as mentioned above, the TLR pathway was suppressed in these patients. In this circumstance, it is reasonable to exclude the belief that TLR recognition may trigger immune bias (Akira et al., 2006). Only B cells but not dendritic cells (DC)/phagocytosis (Mφ) were found to effectively present HBcAg and prime naive T cells (Lee et al., 2009). Farci et al. (2010) further utilized phage display technology to uncover a massive intrahepatic antibody response that was essentially directed exclusively against HBcAg in ALF patients, and the inherently enhanced immunogenicity of HBcAg was demonstrated to be linked to its function as a T cell-independent antigen. Moreover, Chen et al. analyzed the miRNA expression profiles in ALF patients. Their data revealed a distinctive B cell signature with enriched intrahepatic IgM and IgG production exclusively targeting to HBcAg with high affinity. The limited expression of T cell-associated genes, the major negative regulators in ALF patients were found (Chen et al., 2018). Naive-like B cells produced immature IgM through somatic mutation deficiency that has lower cross-reactivation of the original viral Ag. The exaggerated IgM response exclusively leads to binding of the germline antigen to further encounter immune complexes on the surface of infected hepatocytes, resulting in the activation and deposition of C1q, C3, and C4d led to massive liver necrosis (Chen et al., 2018).

Above all, these findings supported the hypothesis that CHB-ACLF appears to be driven by an unusual T cell-independent B cell response inherited from a highly mutated core antigen, leading to complement activation and massive liver necrosis.

## Systematic Inflammation Following DAMP/PAMP Recognition

### Cytokine Storm

Acting in combination, bacterial infection (PAMPs) and liver injury (DAMPs) trigger inflammation to restore tissue homeostasis (Kumar et al., 2011; Kubes and Mehal, 2012). Systemic inflammation that causes severe liver injury through an excessive immune response is a hallmark of ACLF. In contrast to the weak inflammatory response in CHB patients, ACLF patients predominantly secrete inflammatory cytokines or chemokines. Bao et al. (2017) reported that serum IL-6, IL-12, IL-17, IL-23, and TNF-α levels are higher in ACLF patients than in CHB patients. Additionally, ACLF patients have been shown to have high levels of pro-inflammatory molecules such as IL-1β and IL-8 (Claria et al., 2016). The new IL-6/IL-12 family member IL-27 was also higher in ACLF patients than in CHB patients (Zhang G.L. et al., 2014). Colony-stimulating factors that contribute to myelopoiesis, including G-CSF and GM-CSF, were also higher in ACLF patients than in CHB patients and healthy controls. Chemokines such as IP-10 were expressed at a higher level, while others such as RANTS, MCP-1, and MIG were expressed at a lower level in ACLF patients than in CHB patients (Wu et al., 2018). Xin et al. (2016) proposed that six cytokine levels, including hepatocyte growth factor, macrophage inflammatory protein 3α (MIP-3α), carcinoembryonic antigen-related cell adhesion molecule 1, growth differentiation factor 15, E-selectin, and osteopontin, were significantly elevated in CHB-ACLF patients compared to that in CHB patients. This was achieved by detecting cytokine levels in 279 patients with ACLF, 116 patients with CHB, and 20 normal adults. Intrahepatic pro-inflammatory IFN-γ and TNF-α expression was markedly up-regulated in ACLF patients compared to that in CHB and normal controls (Zou et al., 2009). Interestingly, the anti-inflammatory cytokine IL-10 was significantly increased in CHB-ACLF patients, as was the pro-inflammatory IL-6 compared to that in No-ACLF patients. This indicated increased reparative inflammation. Together, these results suggest an excessive host immune response mediated by increased expression of anti-bacterial immunity genes, indicating an increased microbial burden (Wu et al., 2018).

### Immune Cell Dysfunction

Immune cells are thought to be dysregulated during the inflammatory response. In ACLF patients, circulating neutrophil and monocyte counts have been reported to be higher and lymphocyte counts to be lower than those of severe exacerbation of chronic hepatitis B (SE−CHB) patients (Wu et al., 2018). Along with the changes in cell subtype proportions, immune cell phenotypes and functions are also altered.

For example, IL-23 was significantly up-regulated in monocyte-derived dendritic cells (MoDCs) from ACLF patients compared to that in MoDCs from CHB patients in the IT phase (Kubes and Mehal, 2012). The up-regulation of TNF-α in ACLF is positively correlated with increased Kupffer Cells (KCs) (Su and Liu, 1991). The population of peripheral CD14 + CD33 + CD11b + HLA-DR^–/low^ myeloid-derived suppressor cells (MDSCs) in ACLF patients was significantly increased compared to that in CHB patients and healthy controls (Yi et al., 2016; Zeng et al., 2019).

It is well known that natural killer (NK) cells play a crucial role in innate immunity. The percentage of CD3-CD56^+^ NK cells was found to be higher in ACLF patients than in CHB patients. The inhibitory molecule NKG2D was significantly less expressed in NKs in ACLF patients compared to that in CHB patients. In contrast, the activation marker NKG2A was significantly highly expressed in ACLF patients than in CHB and NC patients. These findings indicated an imbalance between NKG2A and NKG2D in NK cells contributing to ACLF progression (Yi et al., 2016). In addition, peripheral NK cells from CHB-ACLF patients expressed higher levels of TNF-related apoptosis-inducing ligand (TRAIL) than those from CHB patients did. TRAIL expression in NK cells was positively correlated with serum IL-6 and IL-8 concentrations in CHB-ACLF patients (Wan et al., 2016). Moreover, expression of other activating receptors such as NKG2D, NKp30, NKp44, and NKp46 was elevated in ACLF patients, but NK cytotoxicity was impaired in patients with ACLF and CHB due to an obvious decrease in cytotoxic CD56^dim^CD16^bright^ NK cells (Liu et al., 2016).

CD8 + T cells were found to be required to control HBV infection through cytolytic and non-cytolytic effector functions (Thimme et al., 2003; Guidotti and Chisari, 2006; Guidotti et al., 2015). Despite no significant difference in the proportion of CD8^+^ T cells in total plasma lymphocytes between ACLF and CHB patients, IFN-γ over-expression and its significant positive correlation with intrahepatic CD8 + and CD4 + T cell accumulation was verified in ACLF patients (Zou et al., 2009; Yi et al., 2016). Increased circulating Th22 inversely correlates with HBV-ACLF prognosis. High Th17 levels in ACLF patients were positively correlated with IL-21 secretion (Mo et al., 2017; Zhang G.L. et al., 2014; Zhang et al., 2018). Th9 cells were unlikely to be involved in HBV pathogenesis, but elevated levels of IL-9 and IL-10 may signal poor ACLF prognosis (Yu et al., 2016).

B cells were also found to be dysfunctional during this period based on increased serum IgA, IgM, and IgG in ACLF patients compared to CHB patients and healthy controls. Furthermore, IgG levels were positively related to IL-27 levels in CHB-ACLF patients. Increased serum immunoglobulins were measured in CHB-ACLF patients (Zhang et al., 2019).

## Immune Exhaustion Accompanied by Bacterial Co-Infection Following the Systemic Inflammation to Organ Failure

### Immune Exhaustion

The host immune system is over-activated during systemic inflammation. However, it is subsequently exhausted at the late ACLF phase. This is strongly supported by the fact that up-regulated inhibitory molecule expression, down-regulated Ag presentation molecules, exhaustion phenotype, and damaged cell function have all been reported in ACLF patients.

The ratio of circulating CD3^+^ T cells to monocytes decreased in ACLF patients beginning at the early to the intermediate stage. This ratio reached the lowest level at the late stage in ACLF, likely due the drastic up-regulation of programmed death-1 receptor (PD-1) in both CD4^+^ T and CD8^+^ T cells, indicating T-cell exhaustion. These CD4^+^ T and CD8^+^ T cells in ACLF patients, along with regulatory T cells, inhibit TNF-α secretion by monocytes (Shi et al., 2010). Moreover, additional B7 super family members have been studied in ACLF patients, including PD-L1 (B7-H1), PD-L2 (B7-DC), B7-H3, and B7-H4. These are highly expressed in liver sections from CHB-ACLF patients (Guo et al., 2012; Cao et al., 2013). Other potential negative regulators such as BTLA are also highly expressed in ALCF patients (Xu et al., 2012). Zhang et al. found KCTD9 to be highly expressed in peripheral and hepatic NK cells from CHB-ACLF patients compared to mild CHB patients. KCTD9 is involved in inhibiting CD69 expression, cytotoxicity, IFN-γ secretion, and inducing a significant decrease in NKG2A receptor expression *in vitro* (Chen T. et al., 2013). Other inhibitory receptors, such as CD158a, on NK cells have also been shown to be increased in ACLF patients (Liu et al., 2016).

Contrary to negative regulators, HLA-DR expression gradually decreased in the monocytes of patients with CHB, liver cirrhosis, and ACLF, especially in the late stage of ACLF compared to that in healthy controls (Xing et al., 2007; Zhang et al., 2016).

### Bacterial Co-infection

It is well known that bacterial infections lead to sepsis and increased mortality in ACLF (Moreau et al., 2013). Spontaneous bacteremia, spontaneous bacterial peritonitis, urinary tract infection, pneumonia, cellulitis, bacterial enteritis, and fungal infection are the most frequent among bacterial infections (Cai et al., 2019). If ACLF is truly a B cell-driven disease that leads to complement activation and massive liver necrosis, DAMPs released from the injured hepatocytes may explain the over-active immune system and the worsened liver injury (Kubes and Mehal, 2012). The liver injury, in turn, leads to the pathological translocation of bacteria or bacterial products, typically originating from the gut floor (Wiest et al., 2014).

**FIGURE 2:**
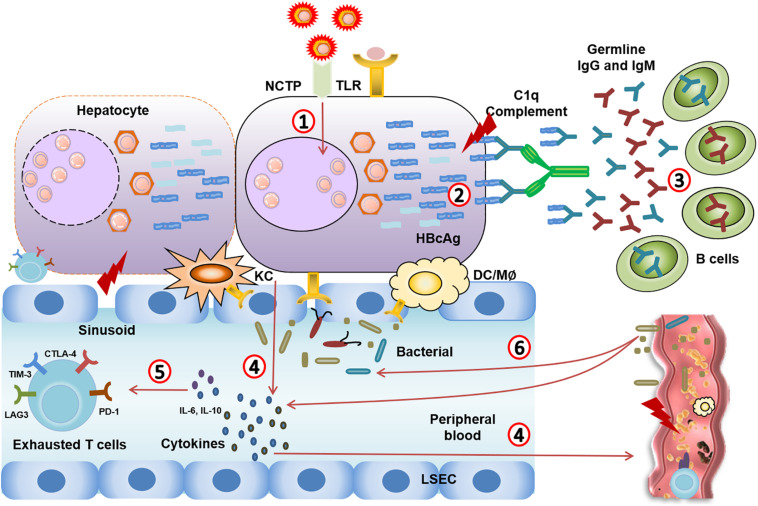
Immune events from CHB to CHB-ACLF. **(1)** HBV virus mutation leads to highly mutated Ags. **(2,3)** Highly mutated core antigens induce overwhelming T cell-independent B cell response, leading to complement activation and massive liver necrosis. **(4)** The damage of hepatocytes induces TLR recognition, immune cells activation, and excessive inflammatory cytokines release in circulating system. **(5)** Systemic inflammation (IL-10, IL-6…) that causes severe liver injury through an excessive immune response, and leads to immune cell dysfunction and immune exhaustion. **(6)** The exhausted immune system leads to the worsening of bacterial or fungal infections. Meanwhile, pathological translocation of bacteria or bacterial products aggregates the systemic inflammation.

Microcirculatory changes in the hepatic and splanchnic vasculature occur at the early stage of ACLF due to immune inflammation (Gronbaek et al., 2012; Laleman et al., 2005). With the exhaustion of the “immune army,” the immune system can no longer fight off infection. The exhausted immune system leads to the worsening of bacterial or fungal infections as endotoxin is released and accelerates disease progression from sepsis to multiple organ failure (Figure 2; Garcia-Tsao and Wiest, 2004; Wiest and Garcia-Tsao, 2005).

## Emerging Early Indicators of ACLF and Potential Therapy Targets

### Early Indicators of ACLF

Even though several biomarkers such as microRNA (hsa-miR-21-5p), the inflammation-mediated protein (N-myc and ATAT interactor, NMI) may be useful novel biomarkers of ACLF (Ding et al., 2015; Xiong et al., 2019), more appropriate, accurate, and early indicators are still needed owing to the rapid progression of ACLF. It is well known that ALCF patients have higher complexity and diversity within the BCP/PC region of HBV, indicating distinct quasispecies characteristics among ALCF patients (Chen L. et al., 2013). ACLF patients infected with HBV show a high frequency of single mutations, including A1762T, T1753V (C/A/G), G1764A, G1896A, and G1899A; double mutations, including G1896A/G1899A and A1762T/G1764A; and triple mutations, including A1762T/C1766T/G1764A and A1762T/T1753V/G1764A (Xiao et al., 2011; Ma et al., 2012). Moreover, HBV mutations such as A1846T is also highly related to the increased risk of ACLF (Nian et al., 2016). These data suggest that BCP/PC mutations play a vital role in ACLF progression and may be useful as early indicators of CHB-ACLF. Beyond viral mutations, more complex factors such as genotypes should also be considered. CHB patients with BCP/PC mutations of genotype B have the high risk of ACLF than patients of genotype C without BCP/PC mutations. In patients of genotype B, A1846T, G1896A, and A1762T/G1764A mutations are more extensive in ACLF patients to CHB patients. Patients of Genotype B with G1896A and A1762T/G1764A are more likely to occur ACLF than patients of genotype C (Ren et al., 2010; Xiao et al., 2011). Thus, on the viral side, specific species and HBV genotypes and variants may be useful early indicators of ACLF. However, clinical data regarding the quantitative and qualitative changes in HBV and the effect of specific genotypes must be collected from ACLF patients and compared to CHB patients in different phases.

On the host side, because many HBcAg mutations have been documented, and their corresponding antibody display is restricted to a VH repertoire lacking somatic mutations, we believe that the detection of HBcAg-targeted unusual humoral immunity may be another potential early indicator for ACLF. Intrahepatic antibodies were targeted to HBcAg exclusively in the germline configuration may present only in ALF individuals (the mutated HBcAg). In chimpanzees with classic acute hepatitis B, no antibodies from the liver were in the germline configuration. In addition, the remarkable affinity of these anti-HBc antibodies is unusual for germline antibodies. And HBcAg is one of the few determined antigens of human germline antibodies, suggesting it to be vital for disease pathogenesis (Chen et al., 2018). Moreover, the significant deposition of complement components C1q, C3, and C4d in the liver tissue of both ALF cases further support a key role for humoral immunity in HBV-associated ALF pathogenesis (Farci et al., 2010). Thus, detecting cell-surface HBcAb and intrahepatic complement components may be a potential early indicator of ACLF. However, these results are from studies on chimpanzee. The dominant B cell signature and exclusive changes of humoral immunity observed in ACLF patients require additional clinical research.

### Therapy Strategies

#### Classical Therapy Strategies

Liver transplantation (LT) is an effective therapy to reduce mortality and increase survival rate (Finkenstedt et al., 2013; Chan and Fan, 2015; Artru et al., 2017). However, the lack of donor organs and high medical cost are major barriers for LT. Non-biological artificial liver support (ALS) devices remove circulating toxins using dialysis-based techniques (Chen et al., 2016; Alshamsi et al., 2020). However, ALS-like MARS provide no significant survival benefit (Banares et al., 2013). Bio-artificial devices that incorporate porcine or human hepatic cells have been developed to replace hepatic detoxification and synthetic functions. But, these devices including extracorporeal liver assist device (ELAD), AMC-BAL bio-reactor, and HepatAssist device have not been tested in ACLF patients and require validation in large studies (Demetriou et al., 2004; Sussman and Kelly, 2014). Anti-viral therapy is required for long-term prognosis. For example, Tenofovir and nucleoside and nucleotide analogs (NUCs) have been reported to improve the transplant-free survival rate in ACLF patients (Garg et al., 2011). However, long-term use of NUCs readily induces acute kidney injury. Peg-IFNα treatment is associated with hepatitis flares. Additionally, glucocorticoids administration in the early phase of HBV-ACLF elevated short-term survival obviously. However, the specifics of GC therapies such as type, dose, and duration should be considered among heterogeneous patients (Fujiwara et al., 2008, 2010; Chen et al., 2014).

#### Advanced Immunotherapy

Granulocyte-colony stimulating factor (G-CSF) has been introduced as a therapeutic alternative to LT in ACLF (Rolando et al., 2000; Garg et al., 2012; Khanam et al., 2014). G-CSF may reduce short-term mortality and prevent worsening of prognosis in mild adverse events (Duan et al., 2013; Chavez-Tapia et al., 2015). Other cell therapies such as bone hepatocyte transplantation, marrow-derived stem cell therapy, mesenchymal/multipotent mesenchymal stromal cell therapy are emerging therapeutic approaches. The most studied cell therapies are mesenchymal stem cells and stromal cells due to they are easy to obtain and able to differentiate into hepatocyte-like cells (Tsolaki and Yannaki, 2015). They can act as regulators of the immune response and repair hepatocyte injury in the recipient (Shi et al., 2017). MSC transplantation has improved liver function and short-term survival in CHB-ACLF patients. However, these studies are lacking of sufficient sample sizes and follow-up periods (Shi et al., 2012; Lin et al., 2017).

**FIGURE 3:**
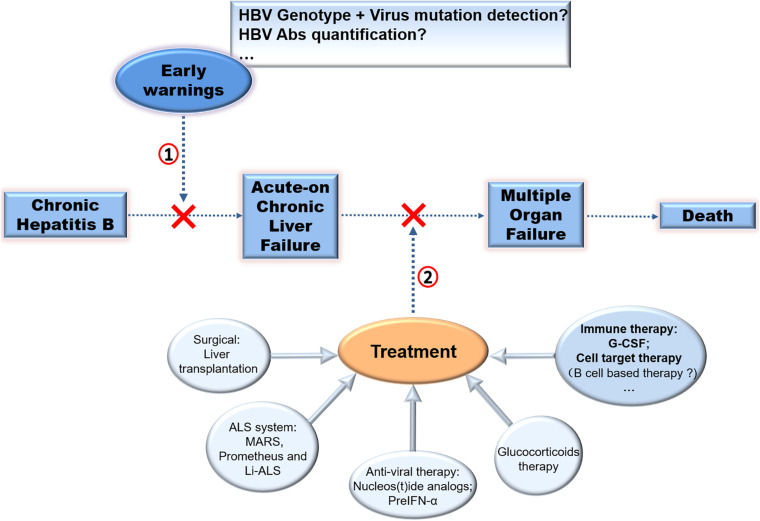
Therapy strategies in CHB-ACLF. Different approaches to prevent disease progression of CHB was exhibited. **(1)** Early warnings of ACLF by detecting virus mutation or HBV Abs quantification, **(2)** treatment of ACLF especially by immuno-based therapy for ACLF require further exploration to improve clinical applications.

In summary, LT is the advised therapy for CHB-ACLF. Cell therapy is an optional method, but it needs validation and has persisting concerns over the long-term safety. Other methods such as NUCs, artificial liver devices, and glucocorticoids could also improve survival in subgroups. Considering the function of host immunity against HBcAg in the pathogenesis of ACLF, it is advised that therapeutic and prophylactic methods for reducing over-activated anti-HBc immune response may be effective. (Figure 3; Farci et al., 2010).

## Conclusion

The imbalanced immune system leads to different HBV progressions, including ACLF. The associated unusual immune events in CHB-ACLF patients have been elucidated. We provide a new understanding of how viral mutations induce immune pressure and how systemic inflammation leads to immune exhaustion culminating in liver damage and multiple organ failure. Additionally, we provide new insights regarding early indicators and immunotherapy for CHB-ACLF. Finally, readily testable early indicators and immuno-based therapy for ACLF require further exploration to improve clinical applications.

## Author Contributions

QL and JW drafted and revised the manuscript. YQ, ML, and HL conceived and designed the manuscript. All authors contributed to the article and approved the submitted version.

## Conflict of Interest

The authors declare that the research was conducted in the absence of any commercial or financial relationships that could be construed as a potential conflict of interest.

## Abbreviations

ACLF: Acute-on-chronic liver failure
ALF: Acute liver failure
CHB: Chronic hepatitis B
BCP: Basal core promoter
CHB-ACLF: Chronic hepatitis B-related ACLF
DCs: Dendritic cells
ELAD: extracorporeal liver assist device
ENH: HBeAg-negative hepatitis
G-CSF: Granulocyte-colony stimulating factor
HBV: Hepatitis B virus
HbeAg: Hepatitis-B-Virus e-Antigen
HBcAg, HBV: capsid/core antigen
IT: Immune tolerant
IC: Immune clearance
IA: Immune active
IRFs: interferon regulatory transcription factors
ISGs: interferon-stimulated-genes
JNK: c-JunN-terminal protein kinase
KCs: Kupffer Cells
LT: Liver transplantation
LPS: lipopolysaccharide
M φ: phagocytosis
MIP-3 α: macrophage inflammatory protein 3 α
MoDCs: monocyte-derived dendritic cells
MDSCs: myeloid-derived suppressor cells
MyD88: myeloid differentiation primary response gene 88
NOD: nucleotide-binding oligomerization domain-containing protein
NK: natural killer
NUCs: nucleotide analogs
PBMCs: peripheral blood mononuclear cells
PAMPs: Pathogen-Associated Molecular Patterns
PRRs: Pathogen Recognition Receptors
PC: pre-core
PD-1: programmed death-1 receptor
SE − CHB: severe exacerbation of chronic hepatitis B
MIP-3 α: macrophage inflammatory protein 3 α
RIG: retinoic acid-inducible gene I
TRAIL: TNF-related apoptosis-inducing ligand
TLR: toll-like receptor
TRIF: TIR-domain-containing adapter-inducing interferon- β
TBK1: TANK binding kinase 1
TAK1: transforming growth factor- β activated kinase 1.
